# Cell wall characteristics during sexual reproduction of *Mougeotia* sp. (Zygnematophyceae) revealed by electron microscopy, glycan microarrays and RAMAN spectroscopy

**DOI:** 10.1007/s00709-021-01659-5

**Published:** 2021-05-11

**Authors:** Charlotte Permann, Klaus Herburger, Martin Niedermeier, Martin Felhofer, Notburga Gierlinger, Andreas Holzinger

**Affiliations:** 1 Department of Botany, Functional Plant Biology, University of Innsbruck, 6020 Innsbruck, Austria; 2 Department of Plant and Environmental Sciences, Section for Plant Glycobiology, University of Copenhagen, 1871 Frederiksberg, Denmark; 3 Department of Nanobiotechnology, University of Natural Resources and Life Sciences Vienna (BOKU), 1190 Vienna, Austria; 4 Department of Biomaterials, Max Planck Institute of Colloids and Interfaces, 14476 Potsdam, Germany

**Keywords:** Cell wall, Conjugation, *Mougeotia*, Sexual reproduction, Streptophyte, Zygospore

## Abstract

*Mougeotia* spp. collected from field samples were investigated for their conjugation morphology by light-, fluorescence-, scanning- and transmission electron microscopy. During a scalarifom conjugation, the extragametangial zygospores were initially surrounded by a thin cell wall that developed into a multi-layered zygospore wall. Maturing zygospores turned dark brown and were filled with storage compounds such as lipids and starch. While *M. parvula* had a smooth surface, *M. disjuncta* had a punctated surface structure and a prominent suture. The zygospore wall consisted of a polysaccharide rich endospore, followed by a thin layer with a lipid-like appaerance, a massive electron dense mesospore and a very thin exospore composed of polysaccharides. Glycan microarray analysis of zygospores of different developmental stages revealed the occurrence of pectins and hemicelluloses, mostly composed of homogalacturonan (HG), xyloglucans, xylans, arabino-galactan proteins and extensins. In situ localization by the probe OG7-13^AF 488^ labelled HG in young zygospore walls, vegetative filaments and most prominently in conjugation tubes and cross walls. Raman imaging showed the distribution of proteins, lipids, carbohydrates and aromatic components of the mature zygospore with a spatial resolution of ~ 250 nm. The carbohydrate nature of the endo- and exospore was confirmed and in-between an enrichment of lipids and aromatic components, probably algaenan or a sporopollenin-like material. Taken together, these results indicate that during zygospore formation, reorganizations of the cell walls occured, leading to a resistant and protective structure.

**Supplementary Information:**

The online version contains supplementary material available at 10.1007/s00709-021-01659-5.

## Introduction

Zygnematopyhceaen green algae have gained much attention as immediate sister group to land plants (Becker 2013; Cheng et al. 2019; Wodniok et al. 2011). With the publication of the first genomes of *Spirogloea muscicola* and *Mesotaenium endlicherianum* (Cheng et al. 2019), new insights into land plant evolution became available. The adaptation to a subareal/terrestrial habitat has particular requirements, and the horizontal gene transfer of e.g. GRAS genes from soil bacteria can be considered as a major step in achieving prerequisites for life on land. The draft genome of *Penium margaritaceum* gives further evidence for additional evolutionary steps necessary for terrestrialization, which includes several classes of carbohydrate active enzymes (CAZy) (Jiao et al. 2020), and reflects *Penium* as a model in cell wall research (e.g. Domozych et al. 2014).

In contrast, the process of conjugation, a characteristic form of sexual reproduction in Zygnematophyceae, and the formation of resistant zygospores and their cell wall composition is only poorly understood. Conjugation involves the fusion of two gametes, which lack organelles for locomotion, and does not directly depend on the availabilty of water (Kadlubowksa 2009; Takano et al. 2019). Conjugation can be performed scalariform (“ladder-like” conjugation), where two different filaments are oriented parallel to each other and fuse their gametes via a conjugation tube; alternatively, it involves two cells from the same filament (Kadlubowska 2009). In the latter case, a distinction between lateral and terminal is made, based on the origin of the conjugation tube. Further distinctions can be made based on the location of gamete fusion and zygospore formation (Kadlubowska 2009). Zygospores are the immediate product of this unique process of sexual reproduction found in Zygnematophyceae. These spores differ in their form, colour and structure, highlighting their importance in species determination (e.g. Stancheva et al. 2013, 2016; Stancheva and Sheath 2012; Takano et al. 2019). The presumed ability of zygospores to endure unfavourable conditions better than asexually formed resting spores is probably found in their multi-layered thick cell wall (Kadlubowska 2009). The complex zygospore wall is composed of three major layers, the endo-, meso- and exospore, which are differentiated by their chemical composition (Simons et al. 1984). While the colourless exo- and endospore are hypothesized to contain cell wall polysaccharides such as cellulose and/ or pectins, the brown or blue coloured interjacent mesospore is frequently ornamented and might consist of algaenan (Poulícková et al. 2007). Algaenan is a resistant biopolymere quite similar to sporopollenin, found in the outer walls of pollen and spores (Montgomery et al. 2016; Poulícková et al. 2007). Unfortunately, the induction of conjugation under laboratory conditions is difficult and was successful only in a few genera like *Spirogyra* sp. (e.g. Takano et al. 2019). It involves a wide range of internal and external factors, making field sampling an important alternative (Gauch 1966; Grote 1977; Simons et al. 1984; Stabenau and Säftel 1989; Yamashita and Sasaki 1979).

In the present study, we selected *Mougeotia* sp. to investigate the process of conjugation, the formation of zygospores and their ultrastructural details in field-collected samples. *Mougeotia* sp. has a long tradition as a cell biological research system and some experiments were groundbreaking for understanding the chloroplast positioning mechanisms, phytochrome research and cytoskeletal involvement in these processes (e.g. Serlin and Roux 1984; Wagner and Klein 1981; Wagner et al. 1972 and references therein). Recently, a transient transformation system was established in *Mougeotia scalaris* (Regensdorff et al. 2018).

In this work, we addressed the following questions, (a) how does sexual reproduction take place in field sampled *Mougeotia* sp. and (b) what is the structural basis for the resistance of zygospores? We addressed this by elucidating the chemical composition of the multi-layered zygospore wall. We hypothesize, that the resistance of these zygospores is related to the chemical composition of the mesospore and that the exospore and the modified conjugation tube have a prominent role during the development of the zygospores. We performed a comprehensive light-microscopical investigation of the conjugation process and zygospore formation by scanning- and transmission electron microscopy (SEM/TEM) to investigate the ultrastructure of the resulting zygospores. We then used Comprehensive Microarray Polymer Profiling (CoMPP) to get insights into the cell wall polysaccharide composition. We furthermore performed a set of cell wall probes to localize abundant cell wall polymers in situ. To get additional insights into the distribution of carbohydrates, lipids, proteins and aromatic components, we applied Raman spectroscopy. With this multi-technical approach, we provide new information for the structural basis for Zygnematophyceaen zygospore resistance.

## Material methods

### Algal material

Algal mats containing Zygnematophyceae were collected at two field sites in the Kühtai valley (Tirol, Austria) in the months May to November 2019/2020. The sampling sites were slow running rivulets or shallow pools of water. In total, 62 samples were taken, of which five contained zygospores or conjugating stages of *Mougeotia* spp. These mats were labelled chronologically by sampling date 1–5 (for additional information see Suppl. Table S1). Mat 1, containing sample 1, grew in a small streamlet near a natural trail (47°19′56″N, 11°09′84″E), while mats 2–5 were located in marshy terrain near the main road leading to the Kühtai village in Tyrol, Austria (47°21′76″N, 11°03′77″E). The collected samples were stored in water taken from the sampling sites at a 16/8 h light–dark regime, 20/15 °C and ~ 30 μmol photons m^−2^ s^−1^ PAR.

### Light-and fluorescence microscopy

Light microscopical investigations of the field sampled *Mougeotia* spp. filaments and zygospores were taken in the following three days after collecting, respectively. The maturation process was observed over a total period of four months, with images taken every 40 days. The analyses were performed using a Zeiss Axiovert 200 M light microscope (Carl Zeiss AG, Jena, Germany), equipped with an Axiocam HRc camera (Carl Zeiss AG, Jena, Germany) and Zeiss Axiovision software. Chlorophyll autofluorescence was visualized with a Zeiss Filter Set 09 (excitation: band pass (BP) 450–490 nm and emission: long pass (LP) 515 nm). A 1% calcolfluor white (Fluka Analytical, Cat# 18,909) staining following the protocol of Herburger and Holzinger (2016) was applied for cellulose and callose detection. The stained cells were than investigated using a Zeiss Filter Set 01 (excitation: BP 359–371 nm and emission: LP 397 nm). For mucilage sheath detection, stainings with Indian ink (Dr. Ph. Martin’s, Oceanside, USA) were performed.

### Scanning-and transmission electron microscopy

Chemical fixation followed the protocol of Holzinger et al. (2006). Briefly, the cells were fixed in 2.5% glutaraldehyde in 20 mM cacodylate buffer (pH = 7) (Fluka, BioChemika, #49,625; Sigma, #C-0125) and embedded in 3% agarose. Then they were post fixed in 1% OsO_4_ (Serva, # 31,251) in 20 mM cacodylate buffer at 4 °C over night and dehydrated in increasing ethanol concentrations and transferred to propylene oxide. Fixed samples where embedded in modified SPURR’s resin (Spurr 1969) (Science Services, #14,300). Ultrathin sections were prepared using a Reichert Ultracut (Leica Microsystems, Wien, Austria), counterstained with 2% uranyl acetate and Reynold’s lead citrate and investigated with a Zeiss Libra 120 transmission electron microscope (Carl Zeiss AG, Oberkochen, Germany) at 80 kV, which was equipped with a 2 × 2 k digital high speed camera and operated by ImageSP software (Albert Tröndle Restlichtverstärker Systeme, Moorenweis, Germany). For scanning electron microscopy, the samples were vapour-plated with gold with a Leica EM SCD050 sputter (Leica Mikrosysteme Ges.m.b.H., Vienna, Austria) and then investigated with a Zeiss EVO 10 SEM (Carl Zeiss AG, Jena, Germany). Due to the risk of sample material loss, the zygospores were directly treated with 70% ethanol instead of a gradual concentration increase for scanning electron microscopy investigations. This procedure did not seem to affect the morphological features of matured zygospores.

### Cell wall extraction and glycan micro array analysis

Field collected *M. disjuncta* samples containing filaments and zygospores were freeze-dried (CoolSafe 15L freeze dryer; LaboGene A/S, Allerød, Denmark) and the alcohol-insoluble residue (AIR) was prepared by washing in 75% ethanol until the supernatant was transparent. Subsequent glycan array analysis (Moller et al. 2007) was done as described in detail before (Kračun et al. 2017). Briefly, cell wall components were sequentially extracted from AIR using 50 mM CDTA (solubilizing pectic compounds) and 4 M NaOH (extracting hemicelluloses). Arrays were printed on nitrocellulose sheets using an ArrayJet Sprint microarray printer (ArrayJet, Roslin, UK). Printed arrays were probed with a set of antibodies (Rydahl et al. 2018; Suppl. Table S2). For controls, primary antibodies were omitted or heat-inactivated prior use. The experiment was repeated three times.

### Immunostaining

*Mougeotia* *disjuncta* zygospores and filaments were immobilized on 12-well slides (ER202W, Thermo Fisher), coated with Vectabond (SP-1800–7, Vector Laboratories) and blocked in 0.5% (w/v) bovine serum albumine (BSA) for 30 min, washed three times in a droplet of Bold’s Basal Medium (BBM) (pH 6.7) for 10 min and incubated with a primary antibody diluted 1:10–1:50 in BBM for 90 min. Samples were rinsed with a syringe spray of BBM, washed (3 × 10 min in BBM) and blocked as described above. Cells were then incubated with a secondary antibody for 90 min in the dark (1:500 in BBM; goat anti-rat IgG (H + L) or IgM, AF488 conjugate (A11006, A21212) or goat anti-mouse AF488 (A28175); Thermo Fisher). After rinsing with a syringe BBM spray and washing (3 × 10 min in BBM), samples were mounted in BBM and antibody binding was visualized using an Olympus BX41 microscope connected to an Olympus U-RFL-T and GXCAM Hichrome-Met AF (FITC filter cube) or with a Leica SP5 confocal laser scanning microscope equipped with an argon (488 nm) laser. For controls, the primary antibody was heat-inactivated prior use (Suppl. Fig. S2 in situ controls).

### Cell wall staining using OG7-13^AF488^ probe

Immunostainings were complemented with the OG7-13^AF488^ probe, which binds long stretches of endogenous demethylated HG via a Ca^2+^-mediated process; the resulting complexes resemble HG ‘egg boxes’ (Mravec et al. 2017a, b). *Mougeotia* *disjuncta* cells were immobilized on Vectabond-coated 12-well slides and probed with 0.5 µl ml-1 OG7-13^AF488^ in BBM (pH 6.7) in the dark. After 60 min, samples were rinsed with a syringe spray of BBM and washed (3 × 10 min in BBM). Samples were mounted in BBM and imaged using an Olympus BX41 microscope (FITC filter cube). For control samples, OG7-13^AF488^ was omitted or replaced by cellohexaose conjugated with AF488.

### Confocal Raman microscopy

Raman imaging of living *M. disjuncta* zygospores (embedded in heavy water (D_2_O)) was performed on a confocal Raman microscope (Alpha300RA, WITec GmbH, Germany). The excitation light source was a linear polarized (0°) coherent compass sapphire VIS laser with λ_ex_ = 532 nm (WITec GmbH, Germany) focused through a 100 × oil immersion objective (numerical aperture = 1.4, coverslip corrected 0.17 mm) (Carl Zeiss, Germany). The backscattered Raman signal was directed through an optic multifiber (50 µm diameter) to a spectrometer (UHTS 300 WITec, Germany) (600 g mm^−1^ grating) and finally to the CCD camera (Andor DU401 BV, Belfast, Northern Ireland). All Raman measurements were taken with a lateral resolution of ~ 330 nm by acquiring at every pixel one full wavenumber spectrum with a laser power of 15 mW and an integration time of 0.18 s. The Control Four (WITec GmbH, Germany) acquisition software was used for the Raman measurements set up. Raman data analysis was performed using the WITec Project Plus 4.1 software (WITec GmbH, Germany). After cosmic ray removal and baseline correction non-negative matrix factorization (NMF) was applied using four to seven endmembers to retrieve the most pure chemical components and visualize their distribution within the area of interest (Prats-Mateu et al. 2018).

## Results

### Morphological investigations illustrate all conjugation stages and reveal major changes in cell content and cell wall structure during maturation

Based on morphological species assignment, sample 1 from sampling site 1 was determined as *Mougeotia parvula*, while samples 2–5, which were all taken from the same sampling site, were determined as *Mougeotia disjuncta*. *Mougeotia parvula* posseses 6–13 µm wide vegetative cells, containing chloroplasts with several pyrenoids arranged in a row (Kadlubowska 2009). Zygospores are spherical in shape with a smooth, brown mesospore, measuring 13–36 µm. The occurance of parthenospores has also been reported in this species in some cases. *M. parvula* has a cosmopolitan distribution, inhabiting sites up to 2360 m a.s.l. It is presumably the most common species of the genus. *M. disjuncta* features vegetative cells with a width of 14–18 µm and chloroplasts with 2–8 pyrenoids arranged in a row. The 24–32 × 21–28 µm zygospores are compressed-globose surrounded by a thick pectic substance. A distinct suture is located on the punctated, chestnut brown surface. This species is mostly known from Europe, the USA (Florida, Fort Meyers) and China (Kadlubowska 2009).

**Fig. 1:**
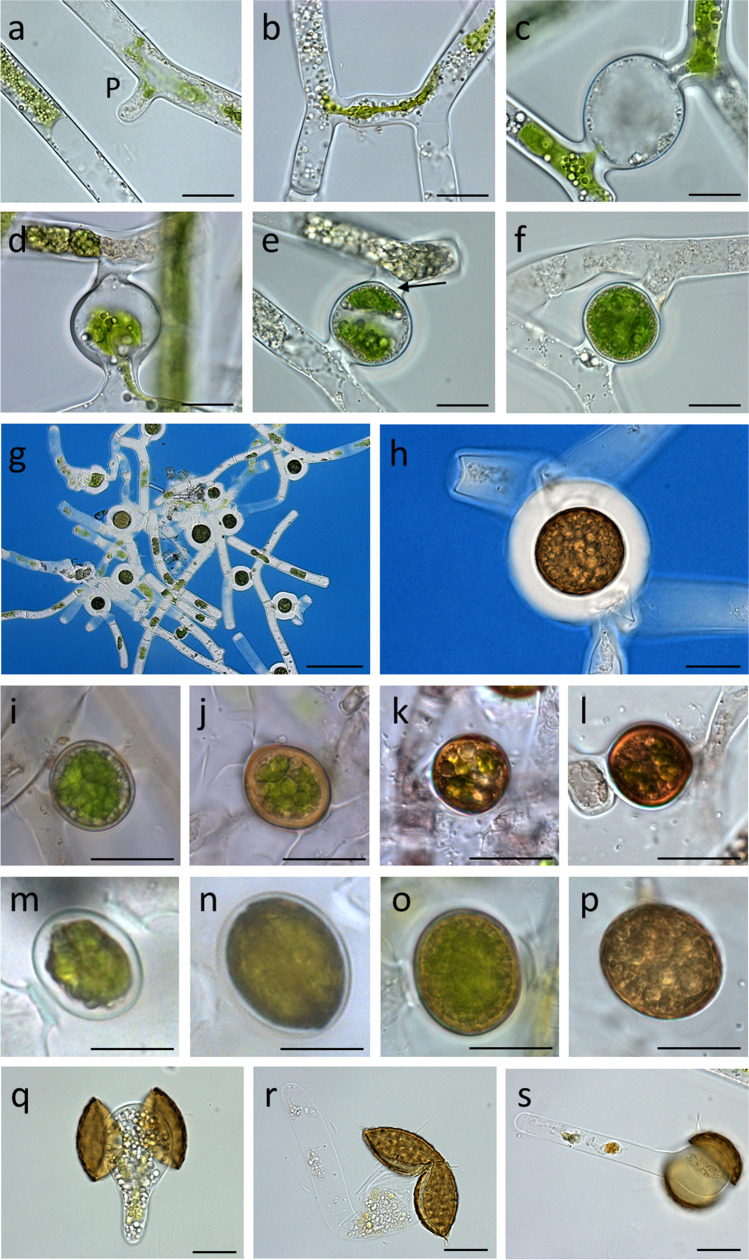
Conjugation stages and zygospores of *Mougeotia* spp*.. M. disjuncta (a-h, m-s), M. parvula (i-l),* conjugation process leading to zygospore formation *(a-f)*, 4-month maturation process *(i-p)*. (**a**) formation of a papilla, (**b**) formation of conjugation tube, (**c**) beginning gamete fusion in conjugation tube, (**d**) gamete fusion, (**e**) zygospore formation final stage (suspensor, formed from closed conjugation tube is indicated by an arrow), (**f**) freshly formed zygospore, (**g**) sheath around zygospores inaccesible for India Ink staining, (**h**) zygospore encased in a gelatinous sheath visualized with India Ink staining, (**i**, **m**) 0 d, (**j**, **n**) 40 d, (**k**, **o**) 80 d, (**l**, **p**) 120 d, (**q**) zygospore wall split in half along suture line, (**r**) germinating zygospore, (**s**) freshly formed filament released from its zygospore. Abbreviation: papilla. Scale bars (a-f, h–s) 20 µm, (g) 100 µm

Sexual reproduction and zygospore formation occurred throughout the entire sampling period (May–September) and was always identified as scalariform isogamous extragametangial. *M. disjuncta* was used to illustrate all conjugation stages, which started with the formation of a papilla (Fig. 1a). The protruding papillae fused between aligned filaments and formed the conjugation tube, resulting in a ladder-like appearance (Fig. 1b). The cytoplasma of both conjugating cells (gametes) migrated into the conjugation tube, where they merged and the zygospore began to develop (Fig. 1c–f). After zygospore formation the closed conjugation tube formed suspensors, keeping the zygospores attached to the filaments (Fig. 1e, arrow). Staining *M. disjuncta* with commercial Indian ink (Dr. Ph. Martin’s Bombay blue) revealed a prominent characterisitc gelatinous sheath (Fig. 1g, h).

The emerging zygospores were enclosed by a thin single-layered cell wall, resembling those of vegetative cells (Fig. 1f, i, m). The zygospores were also still green and showed red chlorophyll autofluorescence (Suppl. Fig. S1a, b). Calcofluor White was used to localize the cell walls of zygospores (Suppl. Fig. S1c, d). During a 4-month maturation process, the zygospores of both species turned yellowish-brown and became surrounded by a thick multi-layered cell wall (Fig. 1i–l M*. parvula*, Fig. 1m-p*M. disjuncta*). An accumulation of storage compounds such as lipid bodies and starch grains was clearly visible in older spores (Fig. 1l, p). The germination process of these matured zygospores was observed in other samples of *M. disjuncta.* The process started with the rupture of the zygospore along the suture line, splitting the wall into two equal parts (Fig. 1q). The two halves where then pressed apart by its growing content and a newly formed vegetative filament was released (Fig. 1r, s).

### *Mougeotia* spp. zygospores differ in their surface ornamentation but coincide in their inner multilayered cell wall structure

**Fig. 2:**
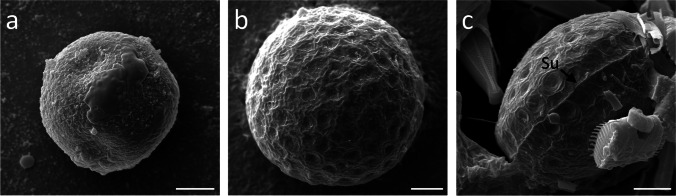
Scanning electron micrographs of *Mougeotia* spp.. (**a**) *M. parvula*, (**b**) *M. disjuncta* zygospore with a micropunctate surface structure, (**c**) *M. disjuncta* zygospore displaying a suture. Abbreviation: Su suture. Scale bars 5 µm

**Fig. 3:**
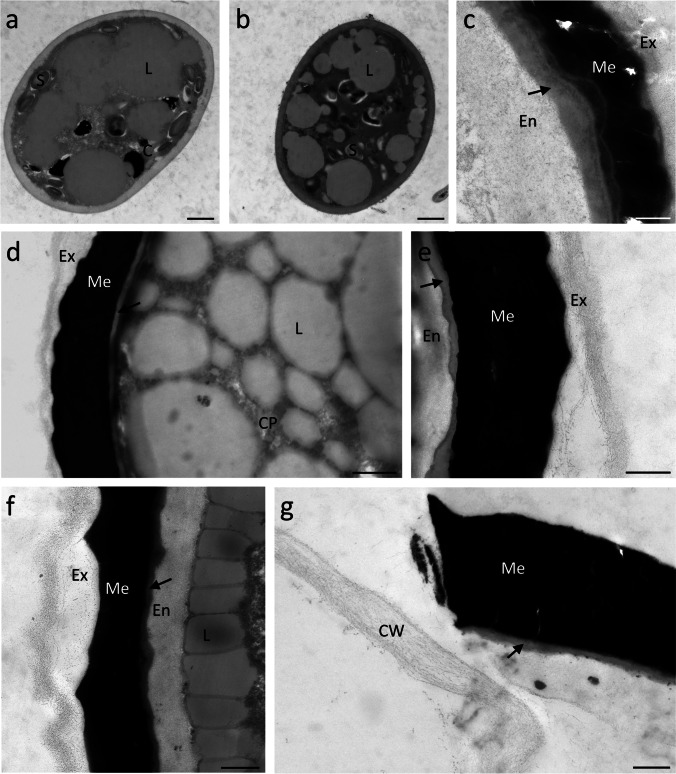
Transmission electron micrographs of *Mougeotia* spp.. *M. parvula (a-c)*, *M. disjuncta (d-g)*. (**a**) young zygospore with single layered cell wall, (**b**) maturing zygospore showing the incipient development of a multilayered cell wall, (**c**) detail view of mature zygospore wall, (**d**) zygospore with lipid bodies spread through cell lumen, (**e**) detail view of mature zygospore wall, (**f**) detail view of zygospore wall with lipid bodies accumulated at the periphery of the cytoplasm, (**g**) pried open zygospore releasing a newly formed filament. Abbreviations: C chloroplast , CP cytoplasma, L lipid body, S starch grain, En endospore, Ex exospore, Me mesospore, arrow indicates a lipid-like fourth layer. Scale bars (a, b, d) 1 µm, (c, e–g) 500 nm

The scanning electron micrographs depicted different surface structures, assisting with morphological species determination. Sample 1, containing zygospores determined as *M. parvula*, showed a round appearance and surface with no exceptional features (Fig. 2a). On the other hand, zygospores of *M. disjuncta* were compressed-globose and characterized by a micropunctate surface structure and a prominent suture (Fig. 2b, c). Transmission electron micrographs of young *M. parvula* zygospores showed large lipid bodies, starch grains, and chloroplast lobes in the cytoplasma as well as a thin polysaccharide-rich cell wall (Fig. 3a)*.* As they matured the incipient development of an additional spore layer was visible (Fig. 3b). Fully developed zygospores of *Mougeotia* spp. displayed the three major layers, endo-, meso-, and exospore (Fig. 3c, e). The outermost thin layer, the exospore, formed a loose, probably amorphous structure on top of the mesospore. The mesospore showed a high electron density and the internal compartmentation in rhomboid blocks was occasionally visible (Fig. 3c). This structure seemed to define the zygospore’s surface ornamentation (Fig. 3f). The inner layer, the endospore, was less electron dense, but appeared different in the two species, being considerably thicker in *M. parvula* (Fig. 3c, e). In both species, a thin lipid-like layer was located between the endo- and mesospore (Fig. 3c–g). The lipid bodies were spread throughout the cell lumen of mature zygospores or accumulated at the periphery close to the endospore (Fig. 3d, f).

### *Mougeotia* exhibits a chemically diverse cell wall architecture

**Fig. 4:**
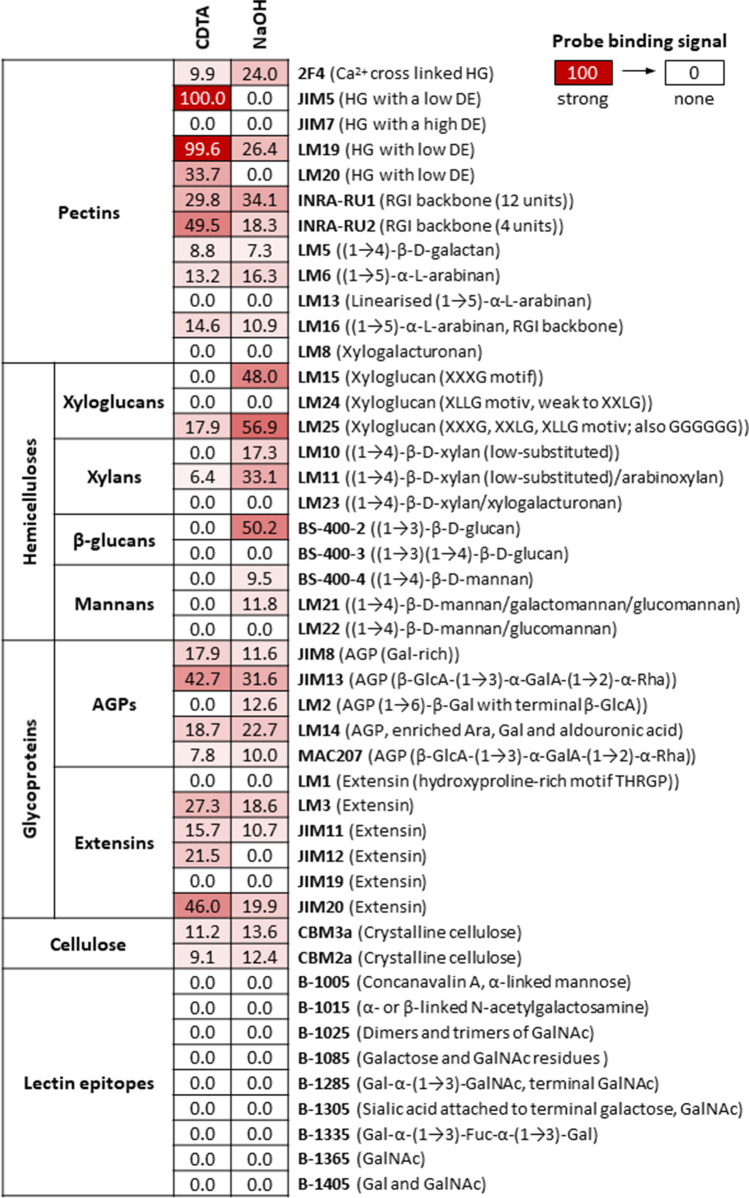
Determining cell wall epitopes in *Mougeotia disjuncta* using carbohydrate array profiling. > 40 cell wall probes were incubated with CDTA and NaOH extracts prepared from *Mougeotia* AIR. The heatmap colour intensity represents the strength of the probe binding and is directly correlated to the numerical value. The strongest signal was assigned value 100 and the cut‐off signal set to 5. Probe codes are in bold and epitopes in brackets. Abbreviations: Ara arabinose, DE degree of esterification, Fuc fucose, Gal galactose, GalA galacturonic acid, GalNAc N-acetylgalactosamin, GlcA glucuronic acid, HG homogalacturonan, RGI rhamnogalacturonan I, Rha rhamnose, AGP arabinogalactan protein. Oligosaccharide nomenclature for xyloglucan probes see Fry et al. (1993)

We subjected field-collected *M. disjuncta* samples to carbohydrate microarray profiling to test for the presence of > 40 cell wall epitopes (Suppl. Table S1). Each sample contained both, filaments and zygospores, which allowed us to gain a comprehensive overview of the algal cell wall composition in the vegetative and the generative stage (Fig. 4). The CDTA fraction was rich in pectin and yielded very abundant binding of JIM5 and LM19 (homogalactauronan (HG) with low DE) and binding of LM20 (HG with higher DE) and INRA-RU1 + 2 (rhamnogalacturonan I (RGI)). Correspondingly, binding was also found for LM16, which recognizes an arabinose-rich side chain of RGI. Furthermore, CDTA extraction yielded glycoproteins as recognized by JIM8, JIM13 and LM14 (arabinogalactan proteins; AGPs), and by LM3, JIM11, JIM12 and JIM20 (extensins). The NaOH fraction was rich in hemicelluloses/β-glucans, yielding abundant binding of probes targeting xyloglucans (LM15, LM25), and callose (BS-400–2). The weaker, yet appreciable binding was found for xylan probes (LM10, LM11) and scarce binding for mannan probes (BS400-4, LM21). Both the CDTA and NaOH fraction yielded a weak CBM2a and CBM3a signal, suggesting the extraction of cellulose. None of the tested lectins produced binding signals.

### In situ cell wall probing suggest a complex cell wall architecture in *Mougeotia*

**Fig. 5:**
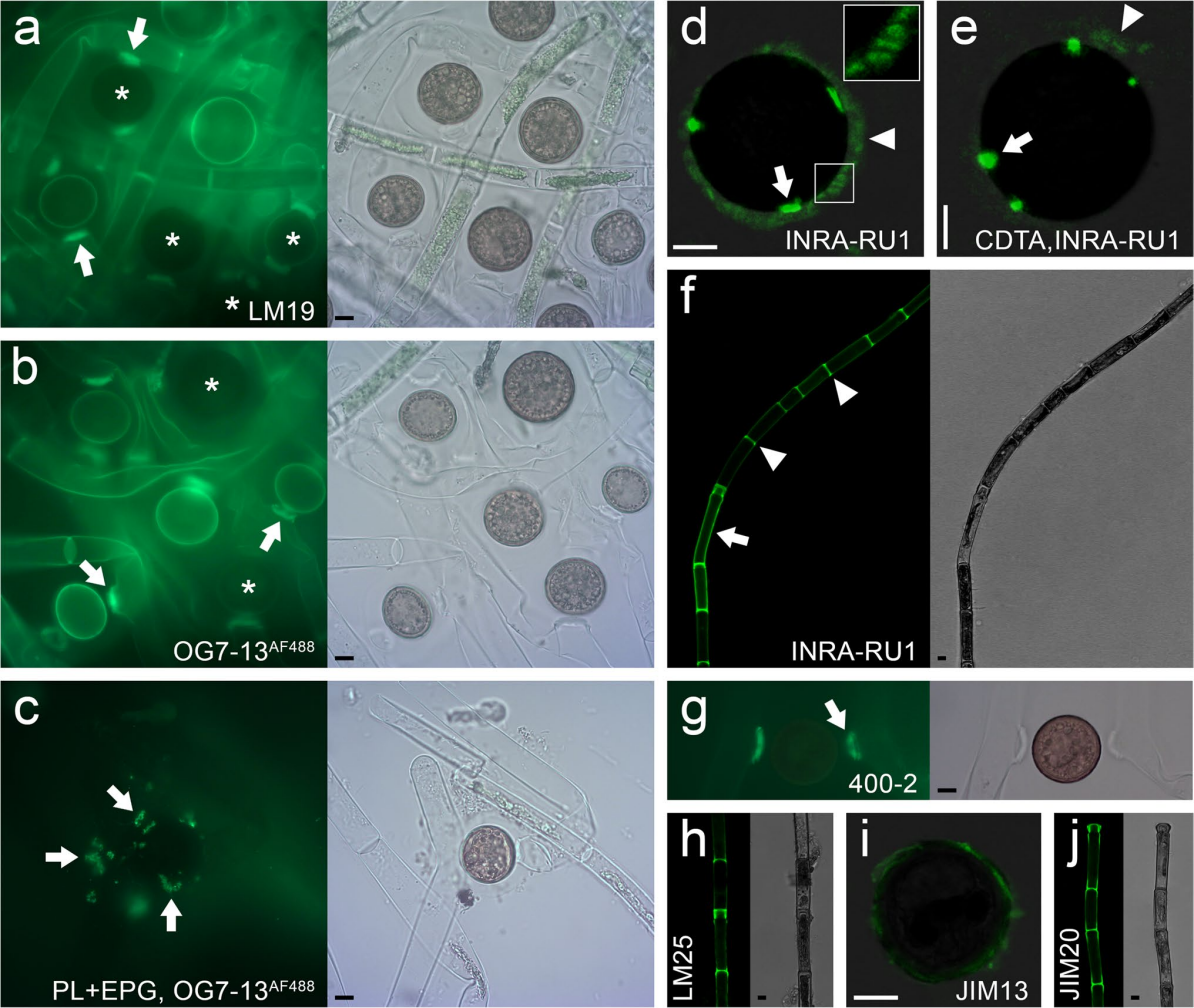
In situ labelling of *Mougeotia disjuncta* cell walls. Fluorescent images (green) are shown on the left and corresponding bright field images on the right *(a-c, f–h, j)* or were merged *(d, e, i)*. Images were taken with an epifluorescence microscope *(a-c, g)* or confocal microscope *(d-f, h-i)*. (**a**) LM19 (recognises HG with low DE) binding to filaments (arrows: suspensor) and zygospores. ~ 50% of zygospores with weak/no signal (asterisks), (**b**) OG7-13^AF488^ (demethylated HG) staining with similar results as in (a): strong staining of suspensor (arrows) and weak signal in some zygospores (asterisks), (**c**) OG7-13^AF488^ staining of cell wall remnants (arrows) after HG removal via PL and EPG, (**d**) INRA-RU1 (RGI) binding to outer sheath (arrowhead) with fibrous structure (inlet) and in inner areas (arrow), (**e**) Some staining in outer sheath after CDTA extraction (arrowhead) close to staining spots in inner wall layer (arrow), (**f**) INRA-RUI (RGI) signal mainly restricted to cross cell walls (arrowheads); occasionally, whole cell wall areas with signal (arrow), (**g**) 400–2 (callose) signal in suspensor, (**h**) LM25 (xyloglucan) signal in cross cell walls, (**i**) JIM13 (AGP) binding in outer zygospore wall with fibrous appearance, (**j**) JIM20 (extensin) binding in areas around cross cell walls. Abbreviations: DE degree of esterification, EPG endo-polygalacturonase, HG homogalacturonan, PL pectate lyase, RGI rhamnogalacturonan I, AGP arabinogalactan protein. Scale bars 10 µm

Based on our carbohydrate microarray profiling experiments, we selected probes that showed strong binding and subjected *M. disjuncta* filaments and zygospores to in situ stainings (Fig. 5). JIM5 and LM19 (recognizing HG with a low DE) delivered the strongest profiling signals. We selected LM19 as a more recently developed HG probe and confirmed abundant probe binding to algal filaments, with a maximum in conjugation tubes (Fig. 5a). Staining was also found in an outer wall layer for ~ 50% of zygospores, indicating different zygospore developmental stages in our sample and a highly dynamic cell wall metabolism in the generative stage. We complemented our antibody staining with another HG probe (OG7-13^AF488^), which binds de-methylated HG and reproduced the LM19 results: Strong binding to filaments and conjugation tubes and to ~ 50% of zygospores (Fig. 5b). OG7-13^AF488^ is a very small probe (oligosaccharide), which allows for a deeper penetration of cellular structures when compared to larger antibodies. However, we did not observe the staining of inner cell wall layers, suggesting that HG is restricted to outer layers and/or the dense cell wall cannot be penetrated by small probes. Subjecting samples to HG digestion with pectate lyase (PL) and endo-polygalacturonase (EPG) prior OG7-13^AF488^ labelling diminished binding signals and staining was restricted to loosely attached cell wall remnants (Fig. 5c). This confirms HG occurs in both filament and zygospore walls.

### Comprehensive microarray polymer profiling shows high abundance of homogalacturonan, xyloglucan, arabino glactan proteins and extensins

Our carbohydrate microarray profiling experiments showed that some of the cell wall probes produced signals in both the CDTA and NaOH fraction (Fig. 4), suggesting that the respective epitopes were tightly integrated into the cell wall and/or part of different cell wall domains. For example, INRA-RU1 (RGI) produced similar signals in CDTA and NaOH fractions (Fig. 4). In situ staining revealed INRA-RU1 binding in (1) an outer zygospore sheath, suggesting the presence of fibrous RGI structures and in (2) highly localized spots inside the sheath (Fig. 5d). Treating zygospores with CDTA prior staining, removed INRA-RU1 epitopes from the sheath, while the highly localized spots inside remained (Fig. 5e). We hypothesize that the highly localized staining sides represent RGI secretion hotspots, which help building up the sheath and thus contain local accumulations of RGI epitopes. This interpretation is supported by the observation that even after CDTA treatment, INRA-RU1 shows some sheath binding close to proposed ‘secretion sites’ (Fig. 5e). INRA-RU1 binding to filaments was heterogeneous. While for most cells, we only found binding to cross cell walls, some cells showed probe binding in both cross- and longitudinal walls (Fig. 5f).

We next tested non-pectin probes, finding strong binding for BS-400–2 (callose) to conjugation tubes (Fig. 5g). In contrast, other filament cell wall areas and zygospores produced very weak signals. The xyloglucan probe LM25 labelled cross cell walls (Fig. 5h). Finally, we examined the glycoprotein probes JIM13 (strongest AGP signal) and JIM 20 (strongest extensin signal; Fig. 4). JIM13 stained the zygospore sheath, producing a fibrous labelling pattern (Fig. 5i), while JIM20 stained areas in and around cross cell walls (Fig. 5j). This suggests that both filaments and zygospore cell walls contain glycoproteins.

### Raman spectroscopy reveals aromatic compounds in the cell wall

**Fig. 6:**
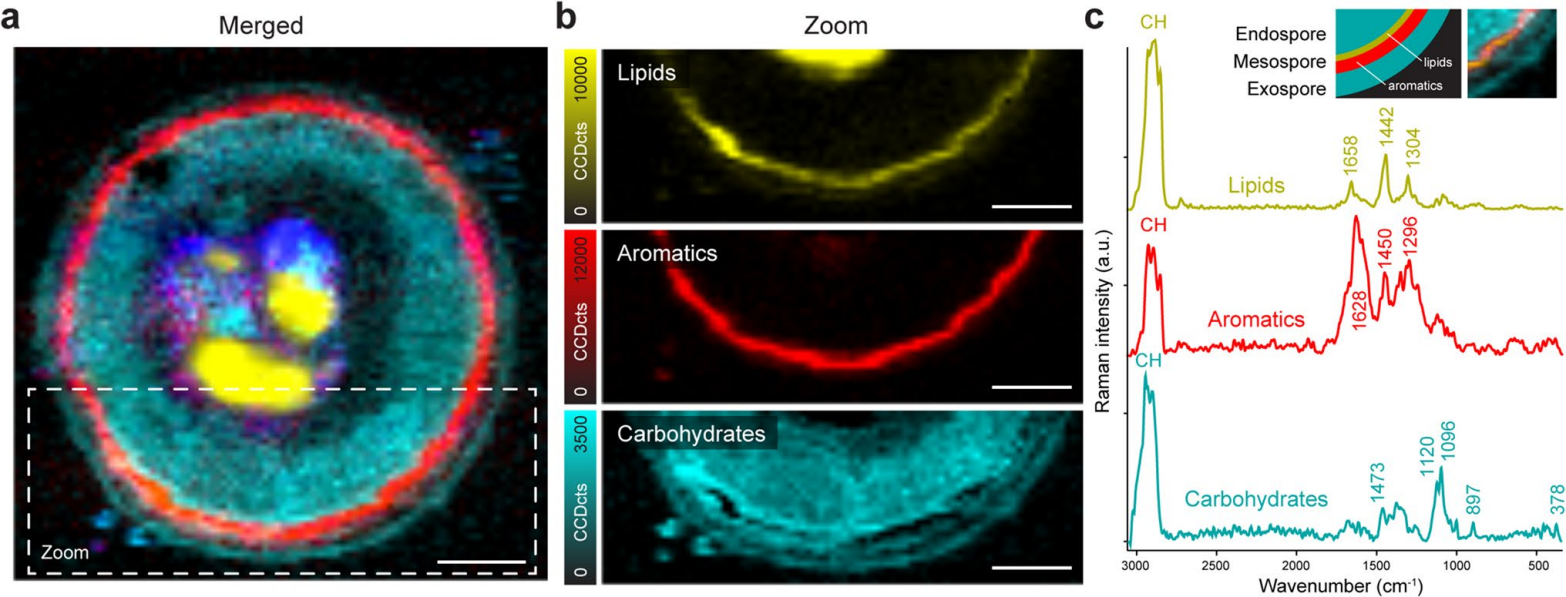
Raman images of *Mougeotia disjuncta* reveal aromatic compounds merged with a lipid layer in the mesospore. (**a**) Merged image of four NMF endmembers including carbohydrates (cyan), lipids (yellow), aromatics (red), and proteins (blue), (**b**) Detailed NMF anlysis of the multi-layered cell wall visualizes lipids, aromatics and carbohydrates, (**c**) Corresponding endmember spectra show respective bands of the three component classes. Inlay represents the overlay of the aromatics and lipids in the mesospore as a schematic drawing and a section of the images from c as an overlay. Scale bars 5 µm

To elucidate the chemical nature of the multi-layered zygospore wall, confocal Raman microscopy combined with advanced multivariate data analysis was performed (Prats-Mateu et al. 2018). Cells were scanned with a spatial resolution of 330 nm (Fig. 6a) and analysed with focus on the outer cell wall (Fig. 6b–c). Non-negative Matrix Factorization (NMF) unmixes proteins, carbohydrates, lipids and aromatics as the main chemical components of the entire zygospore (Fig. 6a and Suppl. Fig. S3). Proteins are represented by an endmember spectrum with protein and nucleic acid bands at 1008, 1459, and 1658 cm^−1^ (Suppl. Fig. S3b) (Rygula et al. 2013) and reflect the chromosomal material mostly in the polar region. Lipids are also found in the centre of the zygospore (Fig. 6a), but also as a continuous layer surrounding the cell (Fig. 6b). The endmember spectrum showed bands at 1658, 1442 and 1304 cm^−1^, which were assigned to lipids (Wu et al. 2011; Prats-Mateu et al. 2016) (Fig. 6c). This layer in the mesospore merged with an aromatic layer, clearly distinguished from each other due to endmembers with different chemical Raman signature (Fig. 6c). The bands at 1628, 1600, 1450 and 1296 cm^−1^ point to an aromatic and lipidic nature (Varsanyi 1969; Prats-Mateu et al. 2016), probably sporopollenin or algaenan (Lutzke et al. 2020; Joseph et al. 2011).The observed high fluorescence in this layer and sensitivity to sample degradation (Suppl. Fig. burnedS3) are typical for aromatic components like lignin (Gierlinger et al. 2012) and sporopollenin (Joseph et al. 2011). Additionally, carbohydrates were visualized as the main components from the endospore to the exospore (Fig. 6b). The corresponding endmember spectrum showed bands for cellulose and hemicelluloses at 1473,1096,1120, 897 and 380 cm^−1^ (Wiley and Atalla 1987, Gierlinger et al. 2012), while the pectin marker band at 856 cm^−1^ (Synytsya et al. 2003, Gierlinger et al. 2012) is absent (Fig. 6c).

## Discussion

In the present study, we have gathered numerous morphological and biochemical data to characterize the sexual reproduction process and the zygospores of *Mougeotia* spp. collected from field samples.

### Species identification using morphological features

The two *Mougeotia* species investigated were determined as *M. parvula* and *M. disjuncta* based on morphological characteristics. Both species perform an isogamous extragametangial scalariform conjugation. While zygospores of *M. parvula* stay attached to their parental filaments, zygospores of *M. disjuncta* are surrounded by a thick pectic layer and the conjugation tube often dissolves during maturation. The two species were furthermore distinguishable based on the mesospore characteristics. *M. parvula* exhibits spherical zygospores, with no distinctive surface structure while *M. disjuncta* forms lenticular zygospores displaying a prominent suture and a punctated mesospore. Both species belong to the section Mesocarpus, which includes species with sexual reproduction and sporangium formation between the two gametangia without their division, as observed in the present study (Gauthier-Lièvre 1965; Kadlubowska 2009). The morphological and cytological features of vegetative filaments allow to identify only the genus. However, the morphological species assignment of Zygnematophyceae is possible based on conjugation characteristics and zygospore properties. In the present study, the most reliable zygospore characteristic was the surface structure of mature cells. In contrast, their size showed a substantial level of variation, as it depends on the total volume of the two conjugating parental cells, which can vary considerably (Czurda 1930; Allen 1958; Poulícková et al. 2007). We observed strong differences in surface structure, whereas the mesospore of both investigated species exhibited a brown coloration. Phylogenetic analyses of the closely related genera *Zygnema* suggested the mesospore color to be an important criterion for *Zygnema* classification (Stancheva and Sheath, 2012).

### Maturation of zygospores

The 4-month maturation process led to the loss of chlorophyll and the formation of a multilayered thick cell wall in both species. Older zygospores accumulated storage compounds (mostly lipid bodies in the centre of the cell) as detected by Raman spectroscopy. The observed changes were similar to those described during the transition of vegetative cells into pre-akinetes in *Zygnema* sp. (Arc et al. 2020). These resting spores, which are still attached to the filament, show a change in chloroplast morphology and ultrastructure, degradation of starch grains and an accumulation of lipid bodies. Metabolite profiling of pre-akinetes showed a re-allocation of photosynthetically fixed carbon into storage instead of growth (Arc et al. 2020). Most likely, similar re-allocation occurred during the investigated zygospore maturation, as TEM micrographs of fairly young zygospores indeed showed chloroplasts and starch grains that degraded during maturation. The lipid bodies were spread throughout the cell lumen or accumulated close to the endospore in the mature state. This peripheral organization is suspected to be part of the maturation process and zygospores with this lipid arrangement may represent an advanced stage.

### Structure and chemistry of the zygospore wall

The mature cell wall of all observed zygospores depicted three major layers and an additional fourth lipid-like layer, which was located between the endo- and mesospore. The endo- and exospore contained various polysaccharides (Poulícková et al. 2007), which was congruent with their loose fibrillar appearance and our detection of carbohydrates in the wall. The Raman signature of the mature zygospore wall confirmed bands attributed to cellulose and hemicellulose, but the pectin marker band at 856 cm^-1^ was not detected (Fig. 6b–c). This means pectin is not or only in very low amounts present within the mature zygote wall. The endospore was denser and more homogeneous than the fairly loose exospore, indicating a different composition and/or structural organization of the components when compared with the exospore. Way less is known about the chemical composition of the intermediate mesospore, which might be the foundation of the zygospores’ remarkable resistance. The mesospore is ornamented, coloured and defines the zygospore surface structure, which is suggested by our TEM investigations and previous studies (Poulícková et al. 2007; Kadlubowska 2009). In mature zygospores, the mesospore exhibits a very high electron density and a “floe-like” inner structure. While earlier studies described a chitinous deposit in the “mesospore”, more recent data suggested lipids and a sporopollenin-like material (Tiffany 1923; Blokker 2000; Poulícková et al. 2007). Using Raman spectroscopy, a mixture of lipids and aromatics is suggested for the mesospore and also a continuous layer of lipids adjacent to the mesospore in both *Mougeotia* species. This structure could represent the lipid-like layer observed in TEM micrographs or support the hypothesis of Poulícková et al. (2007), stating the occurrence of lipids as part of the mesospore. The latter interpretation is supported by our Raman studies which depicted a merge of the lipid layer with a more aromatic layer. The aromatic compounds might reflect a sporopollenin-like material, termed algaenan, as suggested by previous studies (Tiffany 1923; Blokker 2000; Poulícková et al. 2007). Both sporopollenin and algaenan, are resistant biomacromolecules, which differ in their autofluorescence characteristics, location and synthesis (Versteegh and Blokker 2004; Poulícková et al. 2007). Sporopollenin is a chemically highly inert biopolymer found in pollen grains and spores (Kim and Douglas 2013). Despite its importance in gamete protection, its chemical structure was resolved only recently (Li et al. 2019). Sporopollenin likely contains both ester and acetate cross-linkages, contributing to its superior chemical inertness (Li et al. 2019). Based on FT-IR and XPS spectroscopy the sporopollenin in plants was revealed to consist primarily of trans-4hydroxycinnamic and fatty acid, while common methods of sporopollenin isolation result in chemical derivatization (Lutzke et al. 2020). The potential to reveal plant chemistry in situ with FT-IR and Raman spectroscopy becomes clear and continuing research with different treatment (chemical or physical) as well as following development will give further detailed insights into zygospore cell wall cemistry. Algaenans are a series of closely related acid- and base-resistant aliphatic biomacromolecules, whose specific structures are still largely unknown (Tegelaar et al. 1989; Versteegh and Blokker 2004; Kodner et al. 2009). A chemical structure analysis of algaenans from the chlorophytic freshwater algae *Tetraedron minimum*, *Scenedesmus communis* and *Pediastrum boryanum* suggests that it contains long-chain even-carbon-numbered ω9-unsaturated ω-hydroxy fatty acid monomers with intermolecular ester linking and ether cross-linking (Blokker et al. 1998). More recent studies on *Nannochloropsis oculata* (Ochrophyta) confirm previous findings of inter-linked mid-chain alkyl diols as core element of algaenan (Zhang and Volkmann, 2017). While, there is no clear evidence of algaenan in vegetative cells of *Spirogyra, Zygnema* or *Mougeotia*, it was detected in zygospores of *Spirogyra* (Wurdack 1923; Tiffany 1923; Zelibor et al. 1988; Blokker 2000; Versteegh and Blokker 2004; Kodner et al. 2009). This indicates that algaenan might be restricted to the walls of sexually formed zygospores. In this aspect, Zygnematophyceae resemble land plants, where special resistant biomacromolecules such as sporopollenin are absent in most cell types and restricted to spore and pollen walls.

### Compositional changes of *Mougeotia* spp. cell walls during conjugation

The vegetative cell wall compositions of Zygnematophyceae and land plants show considerable similarities (Sørensen et al. 2011; O’Rourke et al. 2015), underlining that land plants inherited cell wall features from their green algal ancestors (Harholt et al. 2016; Jiao et al. 2020). We confirmed that major land plant cell wall components occur in *M. disjuncta*: Microarray profiling, which detects glycan epitopes recognised by antibodies and other probes, verified the presence of HG, RGI, xyloglucans, xylans, callose, AGPs and extensins.

Knowledge on the cell wall composition of Zygnematophyceae during conjugation is scarce. While some studies mainly used unspecific dyes or auto-fluorescence properties (Poulícková et al. 2007; Stancheva et al. 2016) others found binding of lectins to structures emerging during conjugation. For example, the lectins Concanavalin A (CA; binding terminal α-D-mannose and –glucose) and *Ricinus communis* agglutinin (RCA; galactose and N-acetylgalactosamin) strongly labelled papillae and contact zones between papillae in *Spirogyra varians* and *S. castanacea* (Yoon et al. 2009; Ikegaya et al. 2012). Concavalin A strongly labelled papillae of conjugating *Closterium* sp. (Abe et al. 2016). In contrast, papillae in *Zygnema cruciatum* were only weakly labelled by CA and RCA (Kim et al. 2007). The lectin soybean agglutinin (SBA; N-acetylgalactosamin) strongly bound to papillae and contact zones between papillae in *Z. cruciatum* and *S. castanacea*, while binding was absent in *S. varians*. Another lectin, *Ulex europaeus* agglutinin (UEA; L-fucose), bound to vegetative filaments of *Z. cruciatum* but a signal was absent in papillae. In contrast, UEA binding was absent in *S. varians* filaments, but occurred in papillae. This suggests that the cell wall composition of both conjugating structures and vegetative filaments can differ significantly between genera and even between species. Moreover, we tested binding of nine lectins to *Mougeotia* cell walls — including CA, RCA and SBA — but none of them produced appreciable signals. This supports the idea of genera-specific cell wall differences. Interestingly, the above-mentioned authors found binding of lectins to conjugation tubes that recognize N-acetylgalactosamin (RCA, SBA). This suggests the presence of one or more unknown cell wall compounds (glycoprotein and/or poly-/oligosaccharide), which only emerge during the conjugation process. Further studies may mine recently published Zygnematophyceae genomes for the repertoire of glycosyltransferases, potentially discovering new cell wall components that are crucial for sexual processes, as these components might mediate the recognition and adhesion between conjugating filaments (Kim et al. 2007; Abe et al. 2016). This may also address the question, whether small differences in the cell wall composition of papillae as recognized by lectins help avoiding the establishment of conjugation tubes between incompatible species and thus prevent wasting metabolic energy by building non-functional conjugation tubes.

In contrast to *Spirogyra* spp., *Mougeotia* lacks lectin epitopes in conjugation tubes. However, they both contain callose (Fig. 5g; Yamada et al. 2003), corroborating the role of callose in building up dynamic cell wall structures such as pollen tubes (Schoenaers et al. 2017), repair events (Eggert et al. 2014) or cell plates (Verma 2001). We furthermore found abundant binding of HG probes to conjugation tubes and zygospores. HG was detected in the exospore and the stained material was removable by pectate lyase. Furthermore, the outer zygospore layers contained RGI and staining protruded into the gelatinous sheath surrounding the zygospore. The abundant occurrence of charged polysaccharides and cations (e.g. Ca^2+^ mediating HG probe binding) might facilitate hydration of the amorphous outer cell wall layers, all of which might increase the water holding capacity of cells (Herburger et al. 2019) and allow for substrate adhesion (Domozych et al. 2007). Interestingly, outer zygospore wall layers contained AGPs, which are considered key adhesion molecules in Zygnematophyceae (Palacio-López et al. 2019). The capability of zygospores to adhere to their substrate might be important for successful germination within the population, as young filaments emerging from zygospores are sensitive to desiccation stress (Herburger et al. 2019) and airborne germination would bear the risk of exposing filaments to water scarcity and potentially terminate filament growth.

The cell walls of *M. disjuncta* filaments exhibted a complex architecture. While HG probes bound to both cross and longititunal cell walls, RGI was mostly restrited to cross cell walls. We also detected xyloglucan eipitopes in cross cell walls. Xyloglucan epitopes were also found in cross and longitudinal cell walls of *Zygnema* spp. (Herburger et al. 2018). Furthermore, protein extracts prepared form *Zygnema* spp. contained xyloglucan-remodelling enzymes (Herburger et al. 2018). However, unambiguous biochemical evidence for the presence of xyloglucan is still missing in Zygnemtophyceae, despite there is some indication that xyloglucan-like polysaccarides are involved in cell–cell attachement (Ikegaya et al. 2008). This has to be seen crictically as the the presence of the diagnostic isoprimeverose and xyloglucan oligosaccharides releasable by driseleas and xyloglucan‐specific endoglucanases/celluloses, respectively, were not confirmed in Charophytes (Popper and Fry 2003; O’Rourke et al. 2015). This again highlights the relevance of studying a broad range of algal species and genera to gain a more comprehensive insight into algal cell wall architectures.

The present study provides a detailed description of the sexual reproduction morphology of two *Mougeotia* spp. as well as a thorough analysis of the cell wall composition and ultrastructure of the zygospore walls. The multi-layered nature of the cell wall was demonstrated by diverse microscopic techniques. The zygospores were surrounded by a polysaccharide-rich endospore, a massive electron dense mesospore and a thin polysaccharide rich exospore. Between the endo- and mesospore, an additional fourth lipid-like layer was shown by transmission electron microscopy. RAMAN analysis detected aromatic compounds next to an enrichment of lipids in the mesospore, the former most likely representing algaenan or a sporopollenin-like material. Zygospores of different developmental stages contained epitopes of major land plant cell wall components like pectins (e.g. HG), hemicelluloses, (e.g. xyloglucan, xylans) and glycoproteins (AGPs, extensins). In conclusion, these results indicate that the resistant structure of zygospores is attributed to a major reorganization of the cell wall that comprises the formation of special components, not found in vegetative cells.

## Supplementary Information

Below is the link to the electronic supplementary material.

Supplementary file1 (PDF 390 KB)

Supplementary file2 (PDF 137 KB)

## Data Availability

All data that support the findings of this study are available within the article; raw data are available on request from the corresponding author.

## Abbreviations

AGP: Arabinogalactan protein
AIR: Alcohol-insoluble residue
BBM: Bold’s Basal Medium
CA: Concanavalin A
DE: Degree of esterification
EGP: Endo-polygalacturonase
HG: Homogalacturonan
NMF: Non-negative matrix factorization
PL: Pectate lyase
RCA: *Ricinus communis* agglutinin
RGI: Rhamnogalacturonan I
SBA: Soybean agglutinin
UEA: *Ulex europaeus* agglutinin
