# Supplementary material for: Cell wall characteristics during sexual reproduction of Mougeotia sp. (Zygnematophyceae) revealed by electron microscopy, glycan microarrays and RAMAN spectroscopy
Source: Protoplasma. 2021 May 11;258(6):1261–75. doi: 10.1007/s00709-021-01659-5 (PMC8523461; doi:10.1007/s00709-021-01659-5)
Supplement: Supplementary file 1 — Supplementary file1 (PDF 390 KB) [file 709_2021_1659_MOESM1_ESM.pdf]

Supplementary Figures:

Cell wall characteristics during sexual reproduction of *Mougeotia* sp. (Zygnematophyceae) revealed by electron microscopy, glycan microarrays and RAMAN spectroscopy

Protoplasma

Charlotte Permann[+], Klaus Herburger[+], Martin Niedermeier, Martin Felhofer, Notburga Gierlinger, Andreas Holzinger[*]

+ These authors have contributed equally to the study

*Author for correspondence: Andreas.Holzinger@uibk.ac.at
Department of Botany, University of Innsbruck, Functional Plant Biology, 6020 Innsbruck, Austria

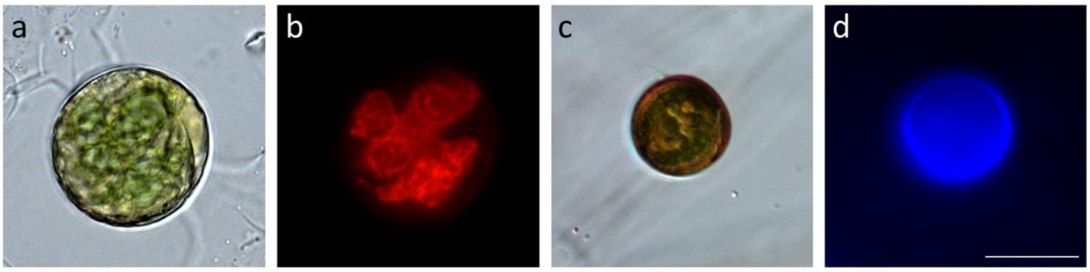

Suppl. Fig. S1 Field sampled zygospores of *Mougeotia*.(a) bright-field image with (b) corresponding red autofluorescence of chlorophyll molecules (Zeiss Filter Set 09 (Excitation: band pass (BP) 450-490 nm and emission: long pass (LP) 515 nm)) in *Mougeotia disjuncta*, (c) bright-field image with (d) corresponding calcofluor white stained image (Zeiss Filter Set 1 (Excitation: band pass (BP) 365/12nm and emission: long pass (LP) 397 nm) in *Mougeotia parvula*. Scale bars 20 µm

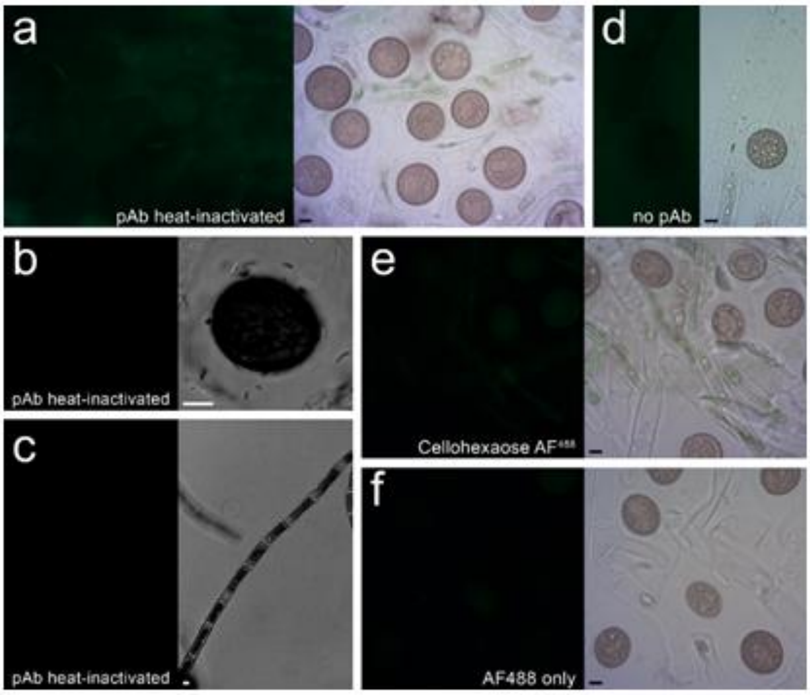

Suppl. Fig. S2 Representative controls of in situ labelling of *Mougeotia* cell walls. Fluorescent images (faint green) are shown on the left and corresponding bright field images on the right. Images were taken with an epifluorescence microscope (a, d-f) or confocal microscope (c, d). (a-d) Heat-inactivating the primary antibody prior use yielded very low florescence signals (AFF488 from a secondary antibody). (d) Omission of the primary antibody produced similar results. (e) Low signal when using cellohexasoe labelled with AF488 instead of the homogalacturonan probe OG7-13AF488. (f) Labelling with AF488 did not give a signal

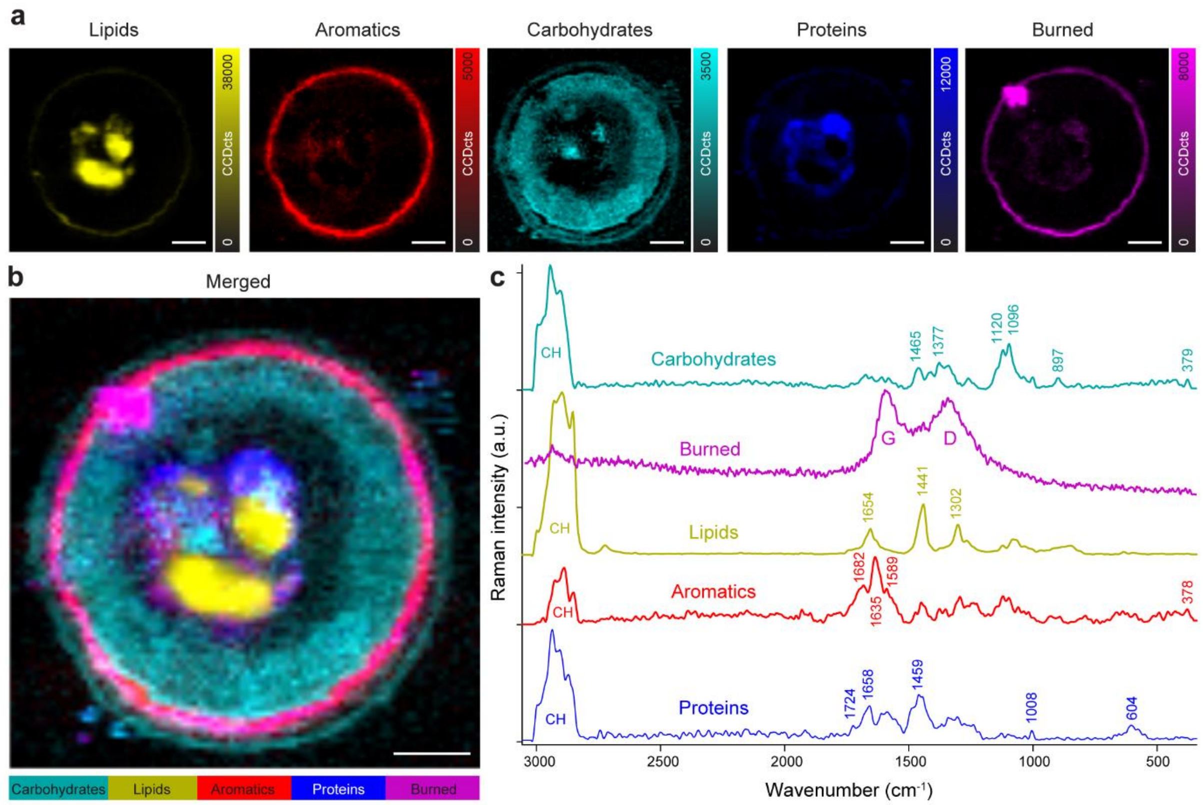

Suppl. Fig. S3 Raman images of the entire cell reveal the different cell content. (a) NMF endmember image display the distribution of lipids (yellow), aromatics (red), carbohydrates (cyan), proteins (blue), and burned area (pink) of the enire cell. (b) Merged image of the five NMF endmembers. (c) Corresponding endmember spectra show bands attributed to carbohydrates, carbon (burned), lipids, aromatics and proteins. Scale bars 5 µm
