# Supplementary material for: Cell wall characteristics during sexual reproduction of Mougeotia sp. (Zygnematophyceae) revealed by electron microscopy, glycan microarrays and RAMAN spectroscopy
Source: Protoplasma. 2021 May 11;258(6):1261–75. doi: 10.1007/s00709-021-01659-5 (PMC8523461; doi:10.1007/s00709-021-01659-5)
Supplement: Supplementary file 2 — Supplementary file2 (PDF 137 KB) [file 709_2021_1659_MOESM2_ESM.pdf]

Supplementary Table:

Cell wall characteristics during sexual reproduction of *Mougeotia* sp. (Zygnematophyceae) revealed by electron microscopy, glycan microarrays and RAMAN spectroscopy

Protoplasma

Charlotte Permann+, Klaus Herburger+, Martin Niedermeier, Martin Felhofer, Notburga Gierlinger, Andreas Holzinger*

+ These authors have contributed equally to the study

*Author for correspondence: Andreas.Holzinger@uibk.ac.at
Department of Botany, University of Innsbruck, Functional Plant Biology, 6020 Innsbruck, Austria

Suppl. Table S1 Sampling dates and coordinates of field samples with zygospores or conjugating stages of *Mougeotia*

Sample Number	Sampling site	Sampling Date	Coordinates	Species
**1**	1	20.07.2019	N47°19'56"<br>O11°09'84"	*Mougeotia parvula*
**2**	2	31.08.2019	N47°21'76"<br>O11°03'77"	*Mougeotia disjuncta*
**3**	2	23.05.2020	N47°21'76"<br>O11°03'77"	*Mougeotia disjuncta*
**4**	2	12.07.2020	N47°21'76"<br>O11°03'77"	*Mougeotia disjuncta*
**5**	2	06.09.2020	N47°21'76"<br>O11°03'77"	*Mougeotia disjuncta*

Suppl. Table S2 Cell wall probes used in the present study

No.	Polymer class	Code	Epitope	Source	Reference
1	Pectin	2F4	Ca2+ cross linked HG	PlantProbes	Liners et al. 1989
2		JIM5	Homogalactauronan with a low DE	PlantProbes	Willats et al. 2000
3		JIM7	Homogalactauronan with a high DE	PlantProbes	Willats et al. 2000
4		LM19	Methylesterified homogalactauronan (low DE)	PlantProbes	Verhertbruggen et al. 2009
5		LM20	Methylesterified homogalactauronan (high DE)	PlantProbes	Verhertbruggen et al. 2009
6		INRA-RU2	Backbone of rhamnogalacturonan I (4 units)	INRA	Ralet et al. 2010
7		INRA-RU1	Backbone of rhamnogalacturonan I (12 units)	INRA	Ralet et al. 2010
8		LM5	(1→4)-β-D-galactan	PlantProbes	Jones et al. 1997
9		LM6	(1→5)-α-L-arabinan	PlantProbes	Willats et al. 1998
10		LM13	Linearised (1→5)-α-L-arabinan	PlantProbes	Moller et al. 2008
11		LM16	(1→5)-α-L-arabinan, RG backbone	PlantProbes	Verhertbruggen et al. 2009
12		LM8	Xylogalacturonan	PlantProbes	Willats et al. 2004
13	Xyloglucan	LM15	Xyloglucan (XXXG motif)	PlantProbes	Marcus et al. 2008
14		LM24	Xyloglucan (XLLG motiv, weak to XXLG)	PlantProbes	Pedersen et al. 2012
15		LM25	Xyloglucan (XXXG, XXLG, XLLG motiv; also GGGGGG)	PlantProbes	Pedersen et al. 2012
16	Xylan	LM10	(1→4)-β-D-xylan (low-substituted)	PlantProbes	McCartney et al. 2005
17		LM11	(1→4)-β-D-xylan (low-substituted)/arabinoxylan)	PlantProbes	McCartney et al. 2005
18		LM23	(1→4)-β-D-xylan/xylogalacturonan	PlantProbes	Pedersen et al. 2012
19	β-glucans	BS-400-2	(1→3)-β-D-glucan	Biosupplies	Meikle et al. 1991

20		BS-400-3	(1→3) (1→4)-β-D-glucan	Biosupplies	Meikle et al. 1994
21	Mannans	BS-400-4	(1→4)-β-D-mannan	Biosupplies	Pettolino et al. 2001
22		LM21	(1→4)-β-D-mannan/galactomannan/glucomannan	PlantProbes	Marcus et al. 2010
23		LM22	(1→4)-β-D-mannan/glucomannan	PlantProbes	Marcus et al. 2010
24	AGPs	JIM8	AGP (Gal-rich)	PlantProbes	McCabe et al. 1997
25		JIM13	β-GlcA-(1→3)-α-GalA-(1→2)-α-Rha)	PlantProbes	Knox et al. 1991
26		LM2	AGP (1→6)-β-Gal with terminal β-GlcA)	PlantProbes	Smallwood et al. 1996
27		LM14	AGP, enriched Ara, Gal and aldouronic acid	PlantProbes	Moller et al. 2008
28		MAC207	AGP (β-GlcA-(1→3)-α-GalA-(1→2)-α-Rha)	PlantProbes	Pennell. et al. 1989
29	Extensins	LM1	Extensin (hydroxyproline-rich motif THRGP)	PlantProbes	Smallwood et al. 1995
30		LM3	Extensin	PlantProbes	Feng et al. 2014
31		JIM11	Extensin	PlantProbes	Smallwood et al. 1994
32		JIM12	Extensin	PlantProbes	Smallwood et al. 1994
33		JIM19	Extensin	PlantProbes	Smallwood et al. 1994
34		JIM20	Extensin	PlantProbes	Smallwood et al. 1994
35	Cellulose	CBM3a	Crystalline cellulose	PlantProbes	Blake et al. 2006
36		CBM2a	Crystalline cellulose	PlantProbes	McLean et al. 2000
37	Lectins	B-1005	Concanavalin A; α-linked mannose	Vector Laboratories	Yue et al. 2009
38		B-1015	Soybean agglutinin; α- or β-linked GalNAc	Vector Laboratories	Yue et al. 2009
39		B-1025	Wheat germ agglutinin; dimers and trimers of GlcNAc	Vector Laboratories	Yue et al. 2009
40		B-1085	Ricinus communis agglutinin; Gal or GalNAc residues	Vector Laboratories	Yue et al. 2009
41		B-1285	Bauhinia purpurea lectin; Gal-α-(1→3) GalNAc, terminal GalNAc	Vector Laboratories	Yue et al. 2009
42		B-1305	Sambucus nigra lectin; sialic acid attached to term. Gal, GalNAc	Vector Laboratories	Yue et al. 2009
43		B-1335	Euonymus europaeus lectin; Gal-α-(1→3)-Fuc-α-(1→2)-Gal	Vector Laboratories	Opitz et al. 2007
44		B-1365	Psophocarpus tetragonolobus lectin; GalNAc	Vector Laboratories	McCarter et al. 2013
45		B-1405	Psophocarpus tetragonolobus lectin II; Gal and GalNAc	Vector Laboratories	Barkhordari et al. 2004

Supporting references

Liners F, Letesson JJ, Didembourg C, et al. (1989) Monoclonal antibodies against pectin: recognition of a conformation induced by calcium. Plant Physiol 91:1419-1424.

Barkhordari A, Stoddart RW, McClure SF, et al. (2004) Lectin histochemistry of normal human lung. J Mol Histol 35:147-156.

Blake AW, McCartney L, Flint JE, et al. (2006) Understanding the biological rationale for the diversity of cellulose-directed carbohydrate-binding modules in prokaryotic enzymes. J Biol Chem 281:29321-29329.

Feng T, Nyffenegger C, Højrup P, et al. (2014) Characterization of an extensin-modifying metalloprotease: N-terminal processing and substrate cleavage pattern of Pectobacterium carotovorum Prt1. Appl Microbiol Biotechnol 98:10077-10089.

Jones L, Seymour GB, Knox JP (1997) Localization of pectic galactan in tomato cell walls using a monoclonal antibody specific to (1 [->] 4)-[beta]-D-galactan. Plant Physiol 113:1405-1412.

Knox JP, Linstead PJ, Cooper JPC, et al. (1991) Developmentally regulated epitopes of cell surface arabinogalactan proteins and their relation to root tissue pattern formation. Plant J 1:317-326.

Marcus SE, Blake AW, Benians TA, et al. (2010) Restricted access of proteins to mannan polysaccharides in intact plant cell walls. Plant J 64:191-203.

Marcus SE, Verhertbruggen Y, Hervé C, et al. (2008) Pectic homogalacturonan masks abundant sets of xyloglucan epitopes in plant cell walls. BMC Plant Biol 8:1-12.

McCabe PF, Valentine TA, Forsberg LS, et al. (1997) Soluble signals from cells identified at the cell wall establish a developmental pathway in carrot. Plant Cell 9:2225-2241.

McCarter C, Kletter D, Tang H, et al. (2013) Prediction of glycan motifs using quantitative analysis of multi-lectin binding: M otifs on MUC 1 produced by cultured pancreatic cancer cells. Proteomics Clin Appl 7:632-641.

McCartney L, Marcus SE, Knox JP (2005) Monoclonal antibodies to plant cell wall xylans and arabinoxylans. J Histochem Cytochem 53:543-546.

McLean BW, Bray MR, Boraston AB, et al. (2000) Analysis of binding of the family 2a carbohydrate-binding module from Cellulomonas fimi xylanase 10A to cellulose: specificity and identification of functionally important amino acid residues. Protein Eng 13:801-809.

Meikle PJ, Bonig I, Hoogenraad NJ, Clarke AE et al. (1991) The location of (1→ 3)-β-glucans in the walls of pollen tubes of Nicotiana alata using a (1→ 3)-β-glucan-specific monoclonal antibody. Planta 185:1-8.

Meikle PJ, Hoogenraad NJ, Bonig I et al. (1994) A (1→ 3, 1→ 4)-β-glucan-specific monoclonal antibody and its use in the quantitation and immunocytochemical location of (1→ 3, 1→ 4)-β-glucans. Plant J 5:1-9.

Moller I, Marcus SE, Haeger A, et al. (2008) High-throughput screening of monoclonal antibodies against plant cell wall glycans by hierarchical clustering of their carbohydrate microarray binding profiles. Glycoconj J 25:37-48.

Opitz L, Salaklang J, Büttner H, et al. (2007) Lectin-affinity chromatography for downstream processing of MDCK cell culture derived human influenza A viruses. Vaccine 25:939-947.

Pedersen HL, Fangel JU, McCleary B, et al. (2012) Versatile high resolution oligosaccharide microarrays for plant glycobiology and cell wall research. J Biol Chem 287:39429-38.

Pennell RI, Knox JP, Scofield GN et al. (1989) A family of abundant plasma membrane-associated glycoproteins related to the arabinogalactan proteins is unique to flowering plants. J Cell Biol 108:1967-1977.

Pettolino FA, Hoogenraad NJ, Ferguson C, et al. (2001) A (1→ 4)-β-mannan-specific monoclonal antibody and its use in the immunocytochemical location of galactomannans. Planta, 214:235-242.

Ralet MC, Tranquet O, Poulain D, et al. (2010) Monoclonal antibodies to rhamnogalacturonan I backbone. Planta 231:1373-1383.

Smallwood M, Beven A, Donovan N, et al. (1994) Localization of cell wall proteins in relation to the developmental anatomy of the carrot root apex. Plant J 5:237-246.

Smallwood M, Martin H, Knox JP (1995) An epitope of rice threonine-and hydroxyproline-rich glycoprotein is common to cell wall and hydrophobic plasma-membrane glycoproteins. Planta 196:510-522.

Smallwood M, Yates EA, Willats WG, et al. (1996) Immunochemical comparison of membrane-associated and secreted arabinogalactan-proteins in rice and carrot. Planta 198:452-459.

Verhertbruggen Y, Marcus SE, Haeger A, et al. (2009) An extended set of monoclonal antibodies to pectic homogalacturonan. Carbohydr Res 344:1858-1862.

Verhertbruggen Y, Marcus SE, Haeger A, et al. (2009) Developmental complexity of arabinan polysaccharides and their processing in plant cell walls. Plant J 59:413-425.

Willats WGT, Limberg G, Buchholt HC, et al. (2000) Analysis of pectic epitopes recognised by hybridoma and phage display monoclonal antibodies using defined oligosaccharides, polysaccharides, and enzymatic degradation. Carbohydr Res 327:309-320.

Willats WGT, Marcus SE, Knox JP (1998) Generation of a monoclonal antibody specific to (1→ 5)-α-L-arabinan. Carbohydr Res 308:149-152.

Willats WGT, McCartney L, Steele-King CG, et al. (2004) A xylogalacturonan epitope is specifically associated with plant cell detachment. Planta 218:673-681.

Yue T, Goldstein IJ, Hollingsworth MA, et al. (2009) The prevalence and nature of glycan alterations on specific proteins in pancreatic cancer patients revealed using antibody-lectin sandwich arrays. Mol Cell Proteomics 8:1697-1707.
